# The use of a technology-assisted and teacher-supervised online discussion platform to promote academic progress in blended embryology courses

**DOI:** 10.1186/s12909-022-03890-x

**Published:** 2022-11-29

**Authors:** Linlin Gong, Yang Song, Yingsong Xu, Mingqi Wang, Haiying Ma, Weiwei Liu, Liang Zhu, Jian Li, Man Luan, Wanjiang Chu, Xiuli Wang, Xin Zhou, Wei Wei, Lihong Hao

**Affiliations:** 1 Department of Histology and Embryology, Dalian Medical University, Dalian, China; 2 Department of Thoracic Surgery, the First Affiliated Hospital of Dalian Medical University, Dalian, China; 3 Institute for Medical Education Research, Dalian Medical University, Dalian, China; 4 College of Basic Medicine, Dalian Medical University, Dalian, China; 5 Department of Anesthesiology, Dalian Medical University, Dalian, China; 6 Academic Affaires Office, Dalian Medical University, Dalian, China; 7 Department of Modern Educational Technology, Dalian Medical University, Dalian, China; 8 Faculty of Applied Sciences, Macao Polytechnic University, Macau, China

**Keywords:** Blended learning, Teacher-supervised engagement, Embryology, Medical education

## Abstract

**Background:**

Students’ engagement with learning materials and discussions with teachers and peers before and after lectures are among the keys to the successful implementation of blended programs. Mixed results have been reported by previous studies on blended learning. This study evaluated the effectiveness of embedding a teacher-supervised online discussion platform in a blended embryology course in terms of its impact on students’ capabilities to handle difficult and cognitively challenging tasks.

**Methods:**

Two forms of blended learning were investigated and compared in this study. Students in the control group (*n* = 85) learned online materials before each class, followed by classroom instruction and activities in which face-to-face discussion and communication between students were encouraged. Students in the experimental group (*n* = 83) followed a similar procedure with an additional teacher-supervised online discussion platform to guide, supervise and evaluate their learning progress. All participants were first-year medical students in clinical medicine at Dalian Medical University who had enrolled in 2017. All participants took the final exam to test their learning outcomes.

**Results:**

The embryology grades of students in the experimental group were significantly higher than those of students in the control group (*p* = 0.001). Additionally, the scores of students in the experimental group on questions with a high difficulty level (*p* = 0.003) and questions assessing high-order cognitive skills (*p* = 0.003) were higher than those of students in the control group; the effect size was moderate (η^2^ > 0.05).

**Conclusions:**

In blended embryology courses, compared with learner-led and face-to-face discussion, the teacher-supervised online discussion platform has great potential to enable students to achieve higher grades and solve difficult and cognitively challenging tasks.

**Supplementary Information:**

The online version contains supplementary material available at 10.1186/s12909-022-03890-x.

## Background

The use of a blended learning approach in the field of medical education is no longer new [1–4]. From the perspective of learners, previous studies reported mixed results in regard to the effectiveness of the blended learning approach [5]. Although most of the evidence in relation to attitudes and preferences appears positive [6], the results are not always significant and in favor of blended learning groups when evaluating learning gains through summative examinations [7]. The reported main challenges include students’ low engagement level with peer discussions and lack of training in self-directed learning strategies.

Very similar patten can be observed in other studies which investigated instructors’ experience in the blended learning environment. Instructors’ attitudes toward the use of blended learning are also mixed. For example, Gray and Tobin’s study [8] reported that instructors have mixed views on the effectiveness of using online communities as a way to promote self-directed learning strategies in a blended learning approach. Jordan [9] found that asynchronous, computer-based instruction was not equivalent to traditional didactics for novice learners of acute care topics. On the other hand, some studies are seemingly more optimistic in using a blended learning approach to minimize the level of supervision by medical instructors, especially in the context where teachers have been traditionally perceived as authority figs [10].

As one of the key components in blended learning environment, the successful use of information technologies is considered as one of the key factors behind the mixed views of teachers and students. However, some instructors reported that they experienced difficulties in integrating these technologies in their teaching, which prevent them from maximizing the benefits of online instructions [11]. These identified factors behind these challenges include instructors’ previous educational and training experience, lack of incentives from school administrative teams, low motivation of using them and low level of self-efficacy in using educational technologies [12]. Moreover, external factors also matter, for example, insufficient support from colleagues and university administration teams, mixed and sometimes vague policies and guidance at both school and national level [13]. Besides teachers’ attitude and the challenges they face, teachers need more experiences applying technology in clinical education in order to respond to current trends towards in the use of technology in tertiary education [14]. Even with a positive attitude to technology, clinical environments have particular challenges. For example, access to computers, time pressures and competing priorities, and the tracking of students’ progress might be an obstacle.

### Problems in medical students’ engagement with online discussions

Most studies have targeted greater use of self-directed learning strategies, and very few have addressed the impacts of interactions and feedback practice in online discussions. It is well known that students’ attention decreases after only 10 min, and students can remember only approximately 20% of the transmitted content directly following a lecture [15]. Therefore, learners’ attitudes and willingness to engage with their peers and the course materials on an online platform are reported to be predictors of learning success [16–19]. Previous studies have found that some instructional designs may promote effective, task-focused peer communication and discussions. For example, in a clinical clerkship program, Koop and others [20] found that students who were required to submit a revised paper demonstrated a much higher level of peer-to-peer interactions and more task-focused discussions on an online platform. Moreover, the introduction of internet-based wiki assignments was also reported to be effective in changing medical students’ use of self-directed learning strategies, such as feedback seeking, peer evaluation and maintenance of learning communities [21].

Other studies have suggested a rather conservative view of the use of peer-to-peer interactions in medical education. Without a high level of supervision and guidance, medical students’ use of online platforms in a blended learning environment has been reported to be less effective; for example, there is a relatively low frequency level of posting messages and communications with peers [22]. The value of teacher presence in organizing, facilitating, and evaluating peer discussions has been confirmed by another study. At a Swedish medical school, researchers found that regardless of learners’ experiences with problem-based learning and gender, teachers’ presence and synchronous communications in peer-based tutorial groups made both cognitive and motivational aspects of the discussion more effective [23]. Moreover, the idea of promoting teacher-learner interactions has been extended to students’ personal online space. For example, Henry’s study [24] reported that the use of a closed Facebook discussion group was overwhelmingly welcomed by students, as it built better rapport and improved perceived learning. In contrast, without instructors’ presence, studies on team-based discussions and learning suggested that students experienced difficulties in identifying certain areas and making suggestions for improvement [25]. As a result, some monitoring of outcomes by faculty members with adequate training have been recommended as a solution.

### Research questions and hypotheses

Bearing in mind the problems of student-led discussions in and the mixed learning outcomes of flipped classes, the purpose of this explanatory study was to assess the effectiveness of a teacher-supervised and structured discussion platform by comparing two groups of embryology students in a blended learning program, and the research questions are as follow: does the engagement with technology-assisted and teacher-supervised online discussion platform 1) promote better academic progress in blended embryology courses? 2) enable learners to perform better in answering difficult questions? and 3) enable learners to perform better in answering questions that assess higher-order cognitive skills? Based on the studies mentioned above, we hypothesize that there is no difference in the learning outcomes between face-to-face discussion and teacher-supervised online guidance in embryology education.

## Methods

### Study settings

The embryology course starts in the second semester of the first year of college. The students participating in this study had a certain ability to learn medical knowledge and had completed a histology course before studying embryology. The blended design of this course was delivered in a national online teaching platform named “MOOCs (massive open online courses) of People’s Medical Publishing House”. This study aims to evaluate the effectiveness in improving students’ learning outcomes of the use of an additional mobile application named “Cloud Class” embedded in the program. Cloud Class is a mobile teaching assistant app launched by Beijing Mosoink Information Technology Co., Ltd. It is a cloud-based service platform dedicated to teaching, specifically aiming to promote interactions between teachers and students before and after classroom instruction. The app can help teachers to supervise a series of learning activities by 1) presenting all the information for students’ self-learning; 2) scheduling learning procedures, such as posting an announcement for MOOC videos, PPT course materials and learning materials before class, providing case-based learning (CBL) cases and exercises during class, and correcting and commenting on brainstormed CBL cases after class; 3) facilitating students in better understanding and applying knowledge through exercises (classroom knowledge absorption) and brainstorming (application of knowledge); 4) guiding discussions about the lectures and brainstorming sessions, helping students review and resolve problems, and providing a platform for instructor-learner communication; and 5) monitoring learning performance according to data feedback and facilitating instructors in providing personalized guidance. At present, this app has been widely implemented in other universities in China to facilitate teaching and learning in a blended learning environment [26, 27].

### Study design

This study design was quasi-experimental with all the participants being assigned to experimental and control group. The main difference lies in that the participants in the experimental group were all trained and invited to participate in a teacher-supervised mobile app-mediated discussion forum. While the learners in the control group organize and discuss similar topics on a face-to-face basis. To answer the research question, the effectiveness of the new mobile technology-supported discussion forum is measured by the difference in the learning outcomes between the two groups one semester after its implementation. The study design of the two groups is shown below (Table 1).

**Table 1:** Study design of the two groups

	Experimental group	Control group
**Before class**	MOOC videos	MOOC videos
	Computer-assisted interactions between instructors and learners, learners and learners	Face-to-face communication was encouraged
**In class**	Lectures were based on CBL, including key and difficult points and lecture-related exercises in the “Cloud Class” app	Lectures were explained by teachers, including key and difficult points
**After class**	Questions were reviewed and resolved through the app; feedback was facilitated by teachers through the app.	CBL cases and lecture-related exercises were provided on paper, and face-to-face communication was encouraged

### Experimental group

#### Before class

In the week before class, the teacher released the course announcement through Cloud Class. Students independently used mobile terminals to learn the online content provided by the MOOC as well as PPT materials, videos and profound knowledge provided by the teachers. Each class was divided into three teams, with approximately 10 students in each team. During the self-learning period, the students could raise questions they did not understand, and the team leaders would organize team members to discuss and summarize the information.

#### In class

**Fig. 1:**
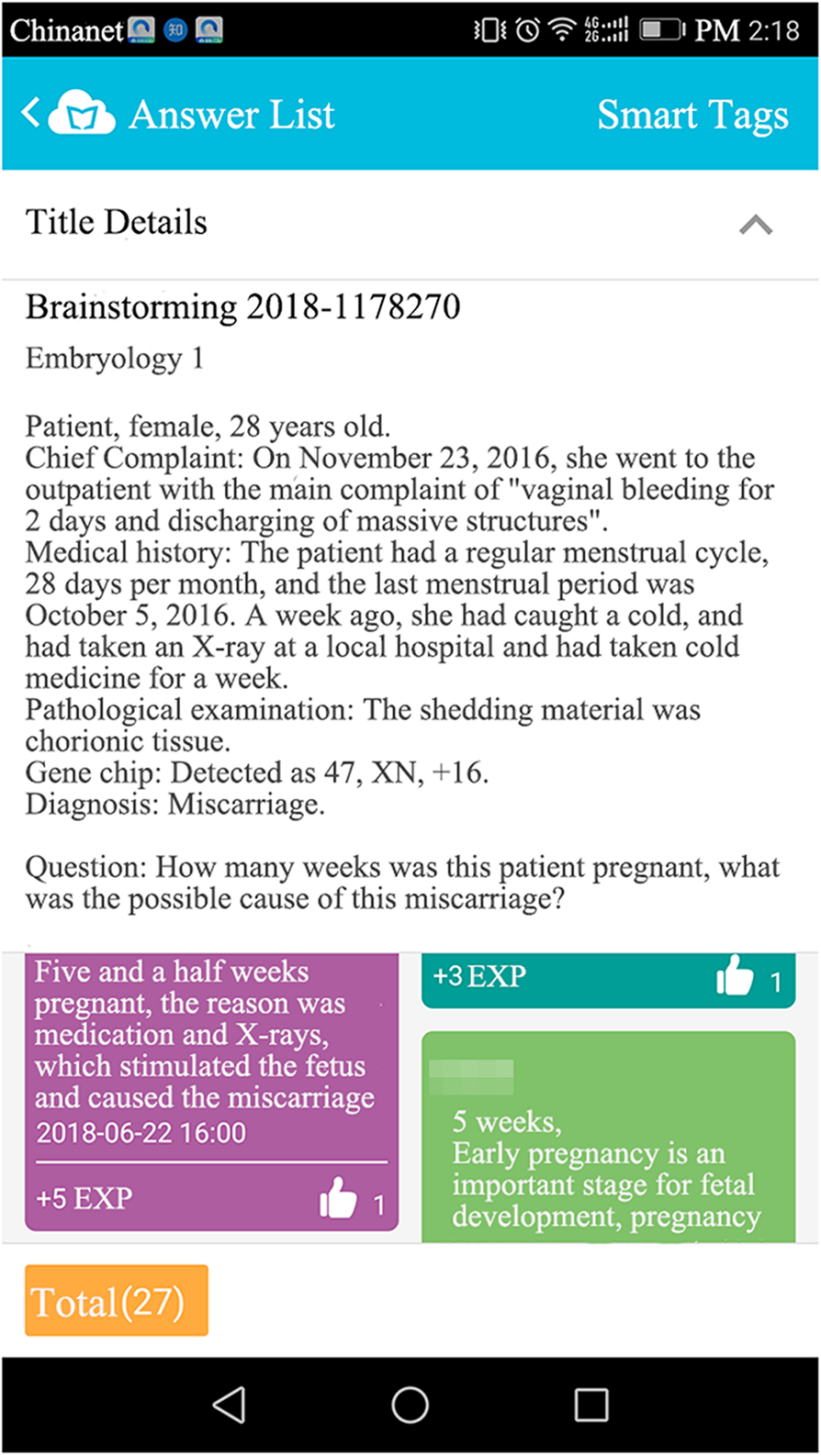
CBL cases were shown in the “brainstorming” module of Cloud Class

**Fig. 2:**
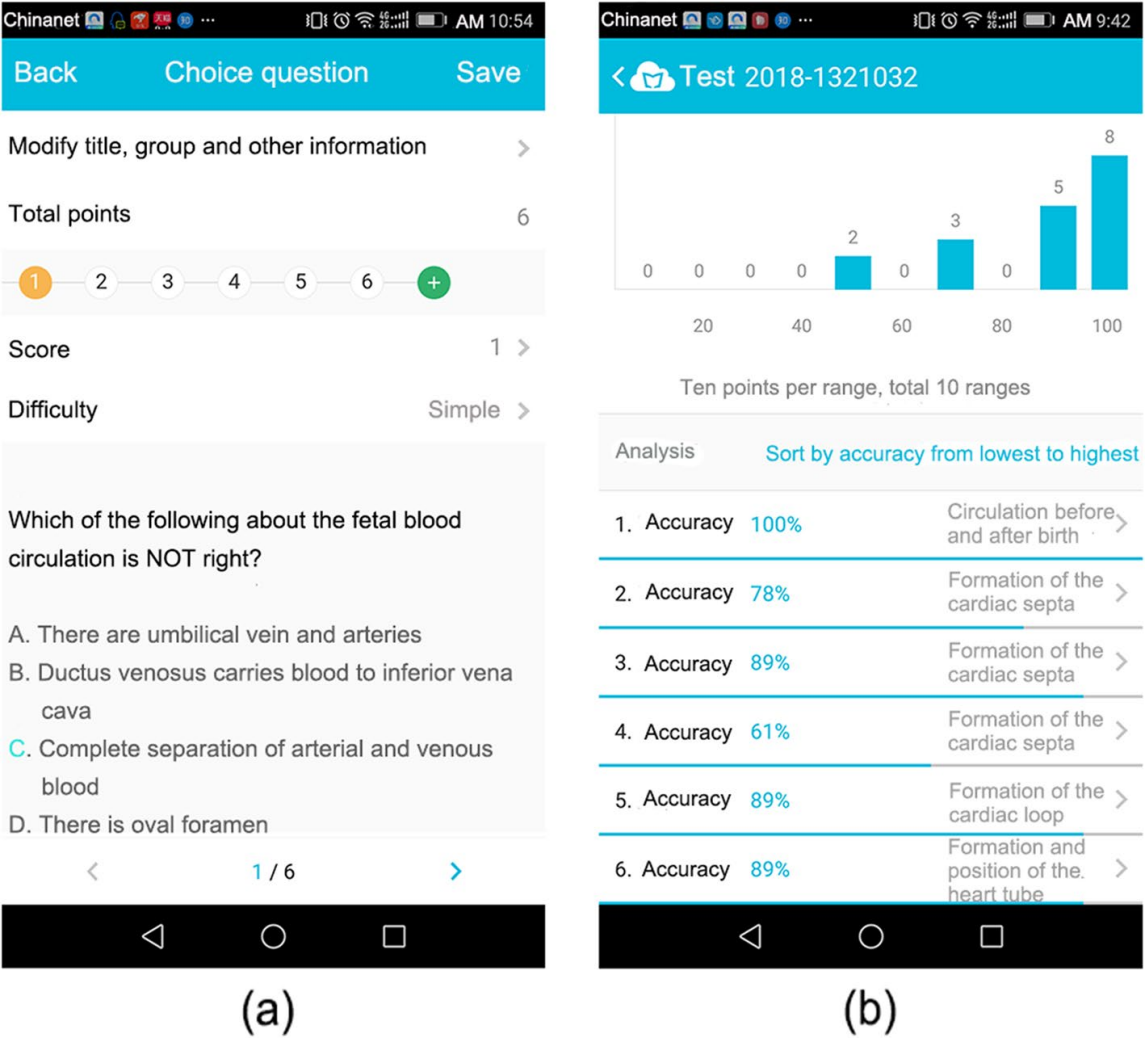
The common choice questions (**a**) and the students’ performance (**b**)

The teachers gave lectures aiming to solve questions and focus on key and difficult points. The team leaders presented the questions raised by team members; then, the teachers rearranged those questions according to the different aspects involved, which assisted students in finding the answers. The teachers issued CBL cases in the “brainstorming” module of Cloud Class (shown in Fig. 1) and arranged student discussions, thereby helping students to conduct self-assessments of their basic understanding of the concepts and clinical applications. At the end of each class, more clinical case exercises were released, and the students were requested to answer questions immediately (as shown in Fig. 2). Based on students’ performance, teachers explained in detail the questions with high error rates.

#### After class

**Fig. 3:**
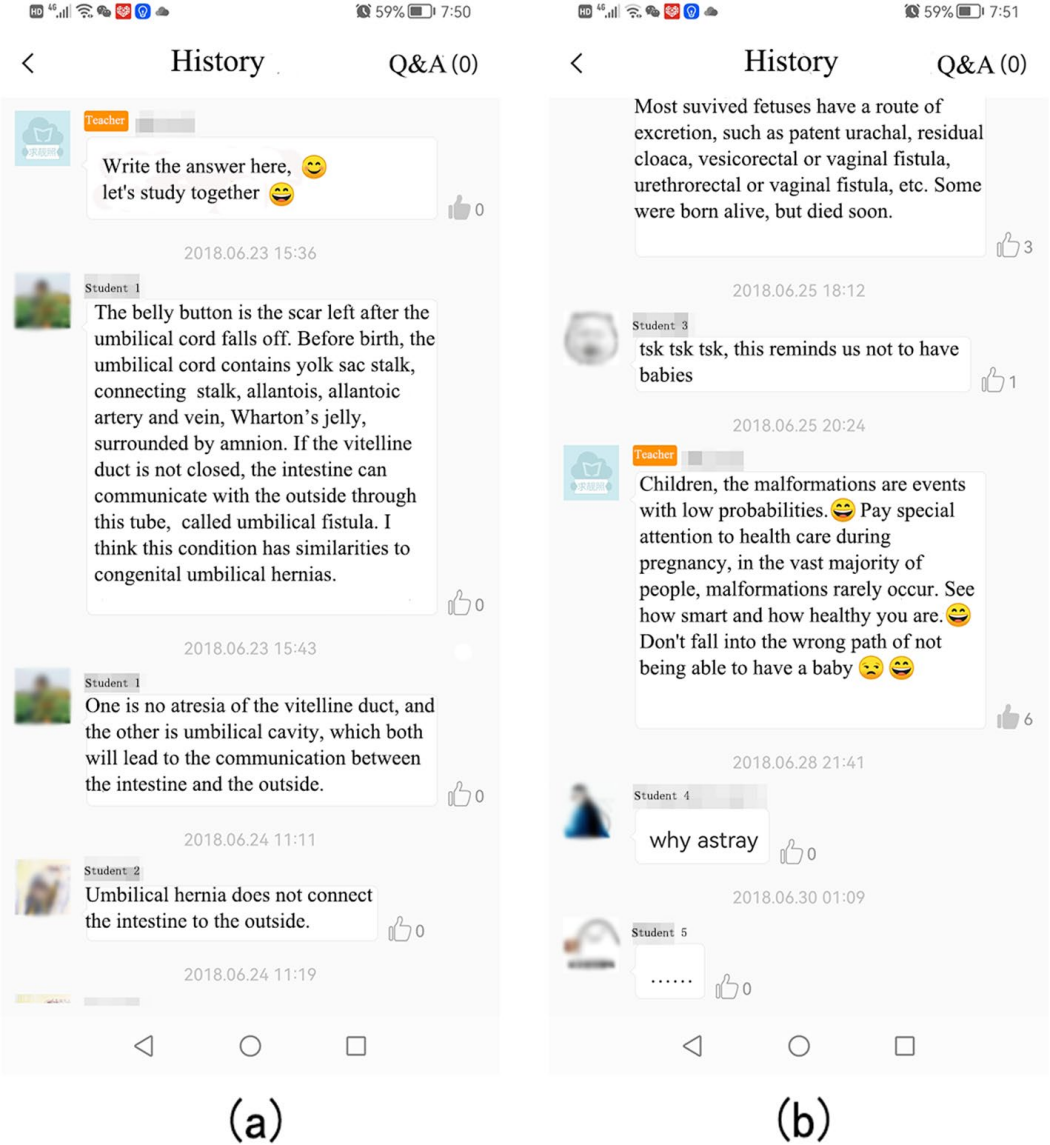
Communication between the instructor and the students

The students reviewed the teaching content through the MOOC and Cloud Class platforms. Further questions were resolved in the Q&A/discussion section. The course arrangement could be adjusted in real time according to the instruction feedback and suggestions collected from the students via the voting/questionnaire function. In summary, the instructors could present tasks and cases (Fig. 1), check students’ performance on quizzes (Fig. 2), and provide feedback and discussion to students (Fig. 3).

### Control group

The difference between the control group and the experimental group was that the control group did not have access to the teacher-supervised online discussion and feedback platform.

#### Before class

As in the experimental group, the teacher released the course announcement and courseware on the MOOC platform, and the students were expected to learn independently with their mobile devices. They were encouraged to talk to each other through their own devices or in a face-to-face condition with no supervision from their teachers.

#### In class

The teachers provided a complete and systematic explanation of the lecture, which is the teacher-led teaching mode in a traditional classroom. However, the depth of the key and difficult points was completely consistent with that for the experimental group. Students were encouraged to raise questions and communicate with teachers in the classroom condition.

#### After class

CBL cases, brainstorming and test parts were provided. As usual, the students were encouraged to ask questions and share their thoughts face-to-face with their teachers or peers.

### Participants

The participants in this study were first-year medical students in clinical medicine at Dalian Medical University who had enrolled in 2017. To avoid the influence of individual teachers, such as their teaching style and personalized teaching materials, six classes of students who had been instructed by teachers from the same research and teaching group were invited to take part in this study. Within the same group, all teachers had collaboratively developed and shared teaching, learning and assessment materials. Among these six classes, totaling 168 students participating in the study, three were selected as the control group, while the other three were selected as the experimental group. The students who were originally selected as participants in the experimental group could voluntarily join the control group at the beginning of the study. As a result, there were 83 students in the experimental group and 85 students in the control group. The age and gender distributions of the two groups of students are reported in Table 2 below. The students participating in this study were provided with all the information about this study, and this study was approved by the Ethics Committee of Dalian Medical University.

**Table 2:** Participant demographics

	Experimental group		Control group	
	Count	%	Count	%
**n**	83		85	
**Age**	19-26 yrs.		19-22 yrs.	
**Mean**	20.5		20.2	
**Sex**				
**Female**	47	56.2	48	56.3
**Male**	36	43.8	37	43.7
**Smartphone ownership**	83	100	85	100

### Description of materials

To answer the first research question, all students participating in this course took a final exam after the course to evaluate their learning gains. The test consisted of eight questions: seven multiple-choice questions and one subjective question. The reliability and validity of the test were evaluated based on answers from 619 students in four previous cohorts who had taken the embryology test. The Cronbach’s alpha coefficient was 0.521, so the test had medium reliability. The validity of the test was still moderate with KMO sampling appropriateness quantity = 0.695 and Bartlett’s test of sphericity: *p* < 0.001; thus, the relevant exploratory analysis could be carried out. To remove the impacts of participants’ previous academic performance on their learning progress in the blended course, their histology course scores from the previous semester were used as a baseline for further comparison.

To answer the second research question regarding whether the difference between the experimental and control group was significantly larger for difficult questions than for easy questions, the difficulty levels of the eight questions on the final examination were predetermined based on the performance of the previous 619 students. Each of the 8 questions in the test paper had its own difficulty coefficient (DC = average score/total score). Among them, the difficulty coefficient of 4 questions (4 points in total) was greater than 0.7; thus, they could be considered relatively simple questions. The difficulty coefficient of the other 4 questions (9 points in total) was less than 0.7; thus, they were considered difficult questions.

To answer the third question regarding whether the difference between the experimental and control group was significantly larger on questions that assessed cognitive skills at a higher level than on those that assessed cognitive skills at a lower level, the cognitive levels of the questions on this test were discussed by all teachers who participated in this study. Guided by the Bloom cognitive competency model, 3 questions on the exam were labeled as assessing “memory”, and 5 questions were labeled as “understanding and application”. The total scores on 3 questions (3 points in total) could be considered low-cognition question scores, while the total scores on 5 questions (10 points in total) could be considered high-cognition question scores.

### Data analysis

The data in this study were all processed using SPSS 23.0. Since most of the data had a non-normal distribution, the Mann-Whitney U test was used for single factor analysis. To eliminate the interference of covariates, the multivariate analysis used ANCOVA and MANCOVA to analyze the original data after rank conversion.

## Results

### Comparison of grades between the experimental group and the control group

The Mann-Whitney U test revealed significant differences in the grades of the two groups, and the overall grades of the experimental group were higher than those of the control group (Table 3).

**Table 3:** Differences in embryology scores

Group	***n***	Average embryology grade (points)	Average rank	Mann-Whitney U test	
				***Z***	***p***
**Experimental**	83	8.36 ± 0.31	96.72	−3.229	0.001
**Control**	85	6.83 ± 0.34	72.57		

### ANCOVA (rank conversion) of embryology grades

The study of histology is a necessary foundation for the study of embryology. We also collected histology grades of two groups and two groups had no significant difference in histology grades (Table 4). This indicated that the two groups of students had the same basis of embryology learning.

**Table 4:** Differences in histology grades

Group	***n***	Average histology grade (points)	Average rank	Mann-Whitney U test	
				***Z***	***p***
**Experimental**	83	40.14 ± 1.09	87.36	−0.752	0.452
**Control**	85	39.62 ± 0.87	81.71		

Using the histology grades as a covariate, ANCOVA was performed based on rank conversion of embryology grades between the experimental group and the control group. The test of homogeneity of variance showed that *p* = 0.201 > 0.05, and there was no interaction between the covariate and the independent variable. The results showed that F = 12.305, *p* = 0.001 < 0.05, estimated marginal average (EMA): experimental group > control group (Table 5), which suggested that the changed teaching mode had a significant impact on students’ overall scores in the final examination.

**Table 5:** ANCOVA (rank conversion) of embryology grades

Group	n	EMA (±SD)	95% confidence interval		F	***p***
			Lower	Upper		
**Experimental**	83	95.145^a^ ± 4.263	86.792	103.561	12.305^b^	0.001
**Control**	85	74.106^a^ ± 4.212	65.789	82.422		

^a^The covariates that appear in the model are evaluated according to the following value: covariate = 84.5

^b^levene’s test of homogeneity of variance: F = 1.648 (*p* = 0.201)

### MANCOVA (rank conversion) of different DC question scores

Whether the questions were simple or difficult, the average scores of the experimental group were higher than those of the control group (simple questions: 3.43 ± 0.77 vs. 2.98 ± 1.11, difficult questions: 4.93 ± 2.45 vs. 3.85 ± 2.47). The scores on simple questions and difficult questions of the experimental group and the control group were converted into ranks, and the histology score was also used as a covariate for MANCOVA. The test of homogeneity of variance showed that *p* = 0.406 > 0.05, and there was no interaction between the covariate and the independent variable. The results showed that for simple questions, F = 6.835, *p* = 0.01 < 0.05, and for difficult questions, F = 9.036, *p* = 0.003 < 0.05 (Table 6), which suggested that the scores of the experimental group were higher than those of the control group regardless of the difficulty of the questions. This effect was more obvious for difficult questions (η^2^ of simple questions and difficult questions were 0.04 and 0.052, respectively).

**Table 6:** MANCOVA (rank conversion) of different DC question scores

Dependent variable	Group	***n***	EMA (±SD)	95% confidence interval		F	***p***	η^**2**^
				Lower	Upper			
**Simple question scores**	**Experimental**	83	92.991^a^ ± 4.562	83.984	101.998	6.835^b^	0.01	0.04
	**Control**	85	76.209^a^ ± 4.508	67.308	85.109			
**Difficult question scores**	**Experimental**	83	93.8^a^ ± 4.346	85.219	102.381	9.036^c^	0.003	0.052
	**Control**	85	75.419^a^ ± 4.294	66.94	83.898			

^a^The covariates that appear in the model are evaluated according to the following value: covariate = 84.5

^b^levene’s test of homogeneity of variance: F = 19.376 (*p* = 0.0001)

^c^levene’s test of homogeneity of variance: F = 0.283 (*p* = 0.596)

### MANCOVA (rank conversion) of scores for high- and low-cognition questions

The average scores of the experimental group were higher than those of the control group (low-cognition question scores: 2.43 ± 0.72 vs. 2.06 ± 0.98, high-cognition question scores: 5.93 ± 2.53 vs. 4.77 ± 2.64). The low-cognition question scores and high-cognition question scores of the experimental group and the control group were converted into ranks, and the histology score was also used as a covariate for MANCOVA. The test of homogeneity of variance showed that *p* = 0.434 > 0.05, and there was no interaction between the covariate and the independent variable. The results showed that in the low-cognition questions, F = 5.352, *p* = 0.022 < 0.05, and in the high-cognition questions, F = 8.945, *p* = 0.003 < 0.05 (Table 7), which suggested that the scores of the experimental group were higher than those of the control group regardless of the type of knowledge. This effect was more obvious in the high-cognition questions (η^2^ of low level and high level were 0.031 and 0.051, respectively).

**Table 7:** MANCOVA (rank conversion) of scores for different types of knowledge

Dependent variable	Groups	***n***	EMA (±SD)	95% confidence interval		F	***p***	η^**2**^
				Lower	Upper			
**Low-cognition question scores**	**Experimental**	83	93.738^a^ ± 4.339	85.171	102.304	5.352^b^	0.022	0.031
	**Control**	85	75.480^a^ ± 4.287	67.015	83.945			
**High-cognition question scores**	**Experimental**	83	92.201^a^ ± 4.676	82.968	101.434	8.945^c^	0.003	0.051
	**Control**	85	76.980^a^ ± 4.621	67.857	86.103			

^a^The covariates that appear in the model are evaluated according to the following value: covariate = 84.5

^b^levene’s test of homogeneity of variance: F = 9.014 (*p* = 0.003)

^c^levene’s test of homogeneity of variance: F = 0.628 (*p* = 0.429)

## Discussion

To answer the research questions, by comparing the academic performance of two groups of students with and without the use of a structured online discussion platform under teacher supervision, this study suggested that the use of discussion under teacher supervision can lead to better learning outcomes in a blended embryology course. More specifically, the effectiveness of this approach is evidenced by 1) better overall scores on the final examination, 2) better scores on questions that assess higher-order cognitive strategies and 3) better scores on questions that have a high difficulty level. In other words, participating in teacher-learner and learner-learner interactions on the discussion platform and observing others’ interactions helped the learners to achieve better learning performance in solving challenging and cognitively complicated tasks. Teachers’ responsibilities on the platform can be summarized as follows: 1) providing all the information for the students’ self-learning, 2) scheduling teaching arrangements, such as posting an announcement for MOOC videos, PPT courseware and learning materials before class, providing CBL cases and exercises during class, and correcting and commenting on brainstorming and CBL cases after class; 3) enabling students to obtain skills and strategies training through the exercises (classroom knowledge absorption) and brainstorming (application of knowledge); 4) guiding discussions about the lectures and brainstorming, helping students review and resolve problems, and providing a platform for instructor-learner communication; and 5) monitoring the learning performance according to the data feedback and providing personalized guidance.

Although studies have linked the idea of peer-to-peer interactions and learning autonomy with better learning outcomes, this study suggested that at least for novice medical students, strong support and a high level of supervision are keys to good performance in a blended learning environment. This finding echoes the results of other studies. For example, previous studies have suggested a long list of barriers that prevent medical students from achieving desirable outcomes in an online learning environment, such as time constraints, absence of institutional strategies and support and negative attitudes [28]. The solutions to all these challenges involve some level of intervention and assistance from the faculty. Moreover, the results of a student-led online discussion forum with limited input from teachers have been found to be superficial and less likely to trigger the use of deep and active learning strategies [29].

Apart from direct communication with teachers, the opportunities of learning from and observing others’ interactions on the teacher-supervised online discussion platform may explain the findings as well. The conventional approach to peer feedback or teacher feedback has addressed the effectiveness of different forms of feedback and comments on receivers’ improvement. However, this study suggested that on a closed team-based discussion platform, learning from others’ mistakes may be another advantage. In some studies, this is also called collaborative reflection [30]. In a flipped learning environment, it is precisely this high level of enthusiasm for engaging with teachers and peers in preclass activities that leads to better performance with more engagement after class [30].

Last, the higher capability of students in the experimental group to handle challenging and difficult examination tasks suggests a link between supervised participation in online discussion and better learning outcomes. This finding supports McLean and others’ claim [31] that the design of flipped classes has great potential to promote students’ deep and active learning strategies. Bearing in mind that the online discussion platform works before the actual classroom discussion and lecturing, the lack of instructors’ assistance in facilitating understanding of the learning materials prior to classes [32] and students’ receptivity to prelecture learning activities [33] may present a real challenge to the success of a blended learning design.

### Implications and limitations

At least two implications can be drawn from this study at both theoretical and practical level. First of all, based on the learning outcomes as measured by summative assessment tasks, teacher presence in technology-assisted discussion appears to be more effective than students’ self-directed discussions in a blended learning environment. It supports the findings from some studies that teachers’ roles in organizing, supervising, evaluating and summarizing peer discussions cannot be underestimated [22, 23]. Secondly, at practical level, the findings of this study highlight the value of teachers’ support of medical students’ self-directed learning in an blended learning environment. Therefore, there is a demand for providing professional training on the development of teachers’ competency in supporting students’ engagement with each other with their electronic devices. Finally, the limitations of this study are twofold. First of all, we could not investigate all factors which may influence medical students’ learning outcomes in a blended learning environment, such as students’ familiarity with mobile learning apps, attitudes towards online learning, previous experiences of using electronic devices to discuss with classmates or teachers. Moreover, we are also aware that students’ demographic factors also play a role, such as their family social economic status, age, and genders. Future studies may consider to collect the information. In addition, a larger sample size and longer-term observations should be completed to fully evaluate the use of teacher-supervised online discussion platform to promote academic progress in blended embryology courses.

## Conclusions

In the blended embryology course, compared with learner-led and face-to-face discussions, the teacher-supervised online discussion platform has great potential to enable students to obtain higher grades and solve difficult and cognitively challenging tasks.

## Supplementary Information

**Additional file 1.**

## Data Availability

All data and materials are available from the corresponding author upon request.

## Abbreviations

MOOCs: Massive open online courses
PPT: Powerpoint
CBL: Case-based learning
KMO: Kaiser-Meyer-Olkin
ANCOVA: Analysis of Covariance
MANCOVA: Multivariate analysis of covariance
